# Supplementary material for: Transcriptomic Insights into Mechanisms of Early Seed Maturation in the Garden Pea (Pisum sativum L.)
Source: Cells. 2020 Mar 23;9(3):779. doi: 10.3390/cells9030779 (PMC7140803; doi:10.3390/cells9030779)

Sprint−2(20)

Global Summary

%DE = 0.79

genes with fdr < 0.2 = 454 (118 + / 336 −)

genes with fdr < 0.1 = 406 (93 + / 313 −)

genes with fdr < 0.05 = 348 (60 + / 288 −)

genes with fdr < 0.01 = 282 (36 + / 246 −)

genes in genesets = 0

<FC> = 0

<t−score> = −58.85

<p−value> = 0

<fdr> = 0.21

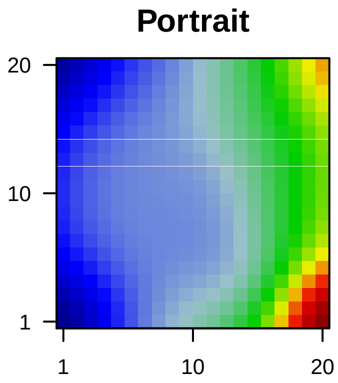

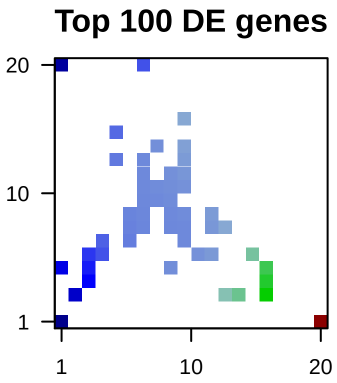

Global Genelist

Rank	ID	log(FC)	p−value	fdr	Metagene	Description
1	comp5786_c0	−0.05	1e−12	1e−10	10 x 16	
2	comp11051_c	−0.02	2e−12	6e−07	11 x 6	
3	TRINITY_DN1	−0.17	5e−09	6e−07	1 x 1	
4	TRINITY_DN4	−0.02	9e−09	2e−06	10 x 7	
5	TRINITY_DN1	−0.07	2e−08	2e−06	3 x 4	
6	TRINITY_DN4	−0.04	4e−08	2e−06	4 x 6	
7	TRINITY_DN2	−0.01	5e−08	2e−06	6 x 7	
8	TRINITY_DN1	−0.03	6e−08	2e−06	4 x 6	
9	TRINITY_DN6	−0.03	9e−08	2e−06	4 x 7	
10	comp22899_c	−0.1	1e−07	2e−06	1 x 5	
11	comp56939_c	−0.02	1e−07	2e−06	10 x 7	
12	TRINITY_DN1	−0.03	1e−07	2e−06	5 x 15	
13	TRINITY_DN9	−0.03	1e−07	3e−06	13 x 8	
14	comp26831_c	−0.02	2e−07	3e−06	10 x 8	
15	TRINITY_DN1	0	2e−07	4e−06	9 x 11	
16	comp16669_c	−0.01	2e−07	4e−06	10 x 7	
17	TRINITY_DN8	−0.67	3e−07	4e−06	1 x 1	
18	TRINITY_DN4	−0.01	3e−07	4e−06	9 x 10	
19	TRINITY_DN3	−0.1	3e−07	4e−06	7 x 20	
20	TRINITY_DN1	−0.18	4e−07	4e−06	14 x 3	

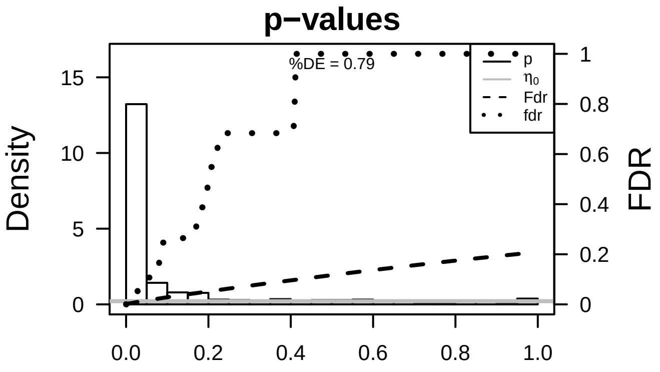

Supplement: Supplementary file 1 [file cells-09-00779-s001.zip › Supplementary materials/FileS3.zip!/FileS2/Hormones - Results/Summary Sheets - Groups/Reports/Sprint.2.20..pdf]
